# The Effects of Supersaturated Hydrogen-Rich Water Bathing on Biomarkers of Muscular Damage and Soreness Perception in Young Men Subjected to High-Intensity Eccentric Exercise

**DOI:** 10.1155/2020/8836070

**Published:** 2020-10-15

**Authors:** Nikola Todorovic, Dejan Javorac, Valdemar Stajer, Sergej M. Ostojic

**Affiliations:** ^1^Faculty of Sport and Physical Education, University of Novi Sad, Novi Sad 21000, Serbia; ^2^Faculty of Health Sciences, University of Pecs, H-7621 Pecs, Hungary

## Abstract

High-intensity eccentric exercise can cause a delayed onset of muscle soreness (DOMS), a short-term condition characterized by muscle damage and tenderness that might hold up recovery and jeopardize exercise routine. Previous studies indicated that hydrogen-rich water (HRW) might be a helpful topical intervention to boost recovery in musculoskeletal medicine, yet no data are available concerning the effectiveness and safety of whole-body bathing with supersaturated HRW after DOMS-inducing exercise. This study evaluates the effects of a single-session bathing with HRW on biochemical markers of muscular damage in healthy young men. The six volunteers who were exposed to DOMS-inducing eccentric exercise were assigned to either supersaturated HRW or control whole-body bathing in a double-blind crossover design. Immediately after an exercise session, the participants were immersed up to the neck into a 200 L bathtub with supersaturated HRW (8 mg of H_2_ per L) or control water (no hydrogen) for 30 min. Blood biomarkers of inflammation and muscular damage and Visual Analogue Scale (VAS) scores for muscle soreness were assessed at baseline (before exercise) and at 24-hour follow-up. Two-way ANOVA revealed a significant difference between two groups in serum creatine kinase (CK) response over the period of intervention (*P*=0.04). A single-session bathing in HRW prevented a rise in circulating biomarkers of muscular damage induced by exercise at 24-hour follow-up, retaining the levels of all biomarkers similar to the baseline values (*P* > 0.05). On the other hand, serum CK, aldolase, and aspartate transaminase were significantly elevated at 24-hour follow-up as compared to the baseline levels after the control bath (342 ± 309 U/L vs. 465 ± 295 U/L; *P* > 0.05). HRW bath also induced a significant drop in VAS scores for muscle soreness in comparison with control water, both immediately after an intervention (32.7 ± 8.6% vs. 20.0 ± 12.8%; *P*=0.02) and at 24-hour follow-up (31.6 ± 24.3% vs. 22.4 ± 27.5%; *P*=0.03), respectively. No participants reported any major side effects during the trial. This pilot study suggests that the whole-body bathing in supersaturated HRW is a safe procedure that attenuates muscular damage and can ease sore muscles after high-intensity eccentric exercise.

## 1. Introduction

Questing after cutting-edge after exercise recovery procedures is a never-ending story. Both recreational and professional athletes are continuously searching for evidence-based strategies that could enable safe, faster, and convenient recovery from exercise [1], allowing the individual to be properly prepared for the next training session(s). Recovering from heavy eccentric exercise appears to be particularly challenging, given the fact that the intensive lengthening contractions are the main cause of muscle damage, known as a delayed onset of muscle soreness (DOMS) [2]. DOMS appears approximately 24–48 h after strenuous eccentric exercise as a consequence of microscopic injuries of contractile proteins accompanied by inflammation and a plethora of muscle-debilitating symptoms and signs. Although DOMS plays an important role in muscle remodeling and favorable adaptations [3], its extended duration may occasionally jeopardize exercise routine and cause more harm than good. This being the case, many approaches have been developed to optimize DOMS recovery, yet the treatment and prevention options remain somewhat limited [4]. Hydrogen-rich water (HRW) has been put forward lately as a worthwhile therapeutics in musculoskeletal medicine. When administered topically to a localized area of the body, HRW improves recovery after ankle sprain [5] and sports-related muscle strains [6]. Hydrogen appears to penetrate the skin easily and is distributed throughout the whole body via the blood in 10 min as measured by hydrogen gas content in expired breath [7]. However, limited information is currently available concerning the potential of whole-body immersion in HRW to ameliorate recovery after high-intensity eccentric exercise. Hence, we investigated, here, the effects of single-session bathing in supersaturated hydrogen-rich water on biomarkers of muscular damage and soreness perception in healthy young men subjected to DOMS-inducing exercise.

## 2. Methods

### 2.1. Participants

Six apparently healthy and active young men (age 24 ± 4 years, weight 80.2 ± 8.7 kg, and height 179.1 ± 8.8 cm) volunteered to participate in this study. All participants signed an informed consent, and the study was approved by the institutional IRB at the University of Novi Sad, with the design systematized in accordance with the Declaration of Helsinki and International Conference of Harmonization Efficacy Guidelines E6. The inclusion criteria for the participants were age from 18 to 30 years, healthy body mass index (e.g., 18.5 to 24.9 kg/m^2^), and no major chronic diseases or acute musculoskeletal disorders. Exclusion criteria included the use of nutritional supplements one month before the study commences. The minimal sample size (*n* = 6) was calculated using the power analysis (G ∗ Power 3.1, Heinrich Heine University, Düsseldorf, Germany), with effects size set at 0.80, alpha error probability at 0.05, and power at 0.80, for two groups and three measurements of study outcomes (correlation among repeated measures 0.5 and nonsphericity correction (*ε*), 1). The primary outcome was the change in the serum levels of creatine kinase (CK) assessed at baseline and at 24 hours after intervention.

### 2.2. Experimental Protocol

The volunteers were allocated in a double-blind crossover design to receive a single-session bath with a highly saturated HRW or control solution (tap water), with each immersion intervention administered after DOMS-inducing exercise. The DOMS-inducing exercise included 5 × 10 eccentric bilateral leg press contractions (120% of one-repetition maximum (1-RM) load) followed by 2 × 10 eccentric bilateral leg press contractions (100% of 1-RM load), with a contraction lasts for 3–5 s, and a subject completed one contraction every 15 s while keeping 3-min rest period between sets. The measurement of 1-RM for each subject was conducted in a familiarization session one week before the experiment, with mean 1-RM load to be 70.8 ± 16.2 kg. The above protocol has been confirmed to induce DOMS in a previous trial [8]. Immediately after completing an exercise bout, the participants were exposed to whole-body immersion. A bathtub solution was produced by dissolving effervescent magnesium-magnesium malate tablets (HRW bath) or nonhydrogen-producing magnesium tablets (control bath) into a 200 L bathtub with tap water, to allow for a whole-body immersion. The bathtub water was thermoneutral (28 degrees Centigrade), and participants were immersed up to the neck and required to sit still in a bathtub for 30 min. Hydrogen gas in HRW solution was produced by the following reaction: Mg + H_2_O ⟶ H_2_ + Mg(OH)_2_, with the concentration of hydrogen in a bathtub to be 8 mg/L as measured by gas chromatography after full dissolution of tablets; a molecular sieve 5 A column and a thermal conductivity detector were used (Hewlett–Packard, 5880 A, USA). HRW and control tablets were formulated to provide an equal amount of magnesium (80 mg per tablet), while control water contained no hydrogen gas. In addition, control tablets produced CO_2_ bubbles that mimic bubbles of hydrogen gas in the HRW solution, making HRW and control baths similar in appearance for effervescence. Both HRW and control bathing tablets are supplied by HRW Natural Health Products Inc (New Westminster, BC, Canada). A wash-out period of 2 weeks was specified to prevent the residual effects of interventions across the study periods. All participants were asked to refrain from using any other recovery procedures during the study. Outcomes assessed at baseline (pre-exercise) and at 24-hour follow-up were blood biomarkers of muscle damage, while Visual Analogue Scale (VAS) scores for muscle soreness were evaluated immediately after exercise, after an intervention, and at 24-hour follow-up. Lab assessments were carried out between 09:00 and 12:00 after an overnight fast; the participants were required to refrain from any exercise 2 days before testing. The venous blood was drawn and centrifuged immediately at 3000 g, with serum separated and analyzed for CK, lactate dehydrogenase (LDH), aspartate transaminase (AST), troponin, and high-sensitive C-reactive protein (CRP) by an automated analyzer (COBAS Pro, Roche, Bazel, Switzerland), while aldolase was evaluated with a photometric method (RX Monza, Randox Laboratories Ltd, Crumlin, UK). Myoglobin was analyzed using an immunoassay system (IMMULITE 1000, Siemens Healthcare GmbH, Erlangen, Germany), and white blood cell count was evaluated using a hematology analyzer (Sysmex XN-L Kobe, Japan). In addition, the participants were instructed to report any side effects of each intervention (e.g., tingling, skin discoloration, burning, itching, and rash) through an open-ended questionnaire.

### 2.3. Statistical Analyses

Two-way mixed model ANOVA with repeated measures was used to establish if any significant differences existed between patients' responses over time of intervention (0 vs. 24 hours). When nonhomogenous variances were identified, values were compared using Friedman's 2-way ANOVA by ranks. The significance level was *P* < 0.05.

## 3. Results

All volunteers completed the course of the study, and no participant reported any major side effects of either intervention. One participant disclosed a localized burning sensation in the skin during the HRW bath when a semidissolved tablet touched the skin, yet this feeling has been described as mild and fleeting. Two-way ANOVA revealed a significant difference between two groups in serum CK response over the time of intervention (*P*=0.04), while no significant differences were found for other biomarkers of muscular damage (*P* > 0.05) (Table 1).

**Figure 1:**
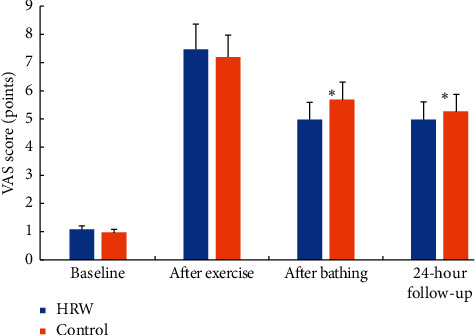
Visual Analogue Scale (VAS) scores for muscle soreness during the study. Asterisk (*∗*) indicates a significant difference between interventions (hydrogen-rich water, HRW vs. control bath) for a change from postexercise VAS scores at *P* < 0.05.

A single-session bathing in HRW prevented an increase in circulating biomarkers of exercise-induced muscular damage at 24-hour follow-up, retaining the levels of all biomarkers similar to the baseline values (*P* > 0.05). On the other hand, serum CK, aldolase, and AST were significantly elevated at 24-hour follow-up as compared to the baseline levels after the control bath (*P* < 0.05). In addition, it appears that high-intensity eccentric exercise significantly increased VAS scores from the baseline to postexercise in both groups (*P* < 0.05) (Figure 1). However, HRW bath induced a significantly superior reduction in VAS scores for muscular soreness in comparison with control intervention, both immediately after bathing (32.7 ± 8.6% vs. 20.0 ± 12.8%; *P*=0.02) and at 24-hour follow-up (31.6 ± 24.3% vs. 22.4 ± 27.5%; *P*=0.03), respectively.

## 4. Discussion

This pilot study demonstrates that a single session of whole-body bathing in supersaturated HRW reduces muscular damage and can ease sore muscles after high-intensity eccentric exercise in young men. HRW appears to be superior to control water whole-body bathing in attenuating the exercise-driven rise in serum CK, implying less local muscular strain and fast-track recovery from soreness when the intervention was administered immediately after exercise. HRW bathing also provokes no major side effects and might, therefore, be put forward as a safe and convenient promising therapeutic modality in sports medicine.

Although often proclaimed as an effective postexercise recovery procedure, the use of whole-body hydrogen bathing is rather rarely scrutinized in scientific studies. In a pioneering placebo-controlled trial, a Japanese group evaluated the effects of 20-minute hydrogen immersion on biomarkers of oxidative stress and DOMS in active young men who performed downhill running for 30 min [9]. The authors found no effects of repeated hydrogen baths on oxidative stress and muscle damage indicators, although VAS scores for DOMS were reduced at 1 and 2 days after an exercise session. Another trial by the same group found no effects of HRW bathing (20 minutes per day for 7 days) on inflammatory markers (including neutrophil dynamics and function) in active men subjected to eccentric exercise [10]. Our study confirmed that hydrogen bath improves VAS scores, yet using supersaturated HRW appeared to effectively prevent any rise in DOMS biomarkers after exhaustive leg press exercise. A longer immersion protocol (30 min) and highly concentrated HRW (8 mg/L) employed in our study perhaps facilitates recovery potential of supersaturated HRW, allowing for the lower-grade release of CK from damaged muscles to the circulation and/or enhanced clearance of CK from the blood, as compared to the control bath. The previous studies suggested that HRW baths did not affect oxidative stress biomarkers [9] and cytokine responses [10], suggesting another possible mechanism(s) that could drive favorable effects of HRW on muscle soreness. Hydrogen could affect gene expression [11], but this effect perhaps appears in slow dynamics, while HRW bathing manifests biological effects comparatively rapidly. Hypothetically, HRW bathing could maintain sarcolemma intact (or less permeable) to enzyme leakage by stabilizing muscle cell viability via acute modulation of central nuclei-related mechanisms [12]. This possibility should be explored in forthcoming studies.

Elevated circulating CK after exercise is a key indicator of muscle cell disruption, with the highest postexercise serum enzyme activities found after prolonged exercise that includes eccentric muscular contractions. Serum CK concentration peaks at 24 h after DOMS-inducing exercise and then decreases approximately 35% per day to baseline levels [13]. In general terms, a training break is suggested to athletes who experience high CK after exercise in aim to enable myocyte regeneration and remodeling. However, a prolonged hiatus may disrupt a dense exercise schedule in many professional sports that require a day-to-day training practice. Therefore, any intervention that enables faster recovery after strenuous exercise in terms of keeping serum CK equilibrium might be of high importance for professional and recreational athletes with high exercise load. We found that a single-session bathing with supersaturated HRW prevented the upturn of serum CK concentrations induced by eccentric resistance exercise and therefore may facilitate postexercise recovery. However, does HRW bathing interfere with muscle remodeling and favorable adaptations after DOMS-inducing exercise remains to be addressed in future. Interpreted from the degree of CK elevation found in the present study, it appears that only slight muscle damage occurred during this exercise regimen. In addition, the baseline values of CK appear to be slightly higher than the reference values, perhaps due to the fact that we recruited physically active volunteers that often have elevated CK levels [14]. Although the participants were required to refrain from exercise 48 hours before testing in the present study, a longer absence from exercise might be required to taper CK levels in this population [15] before an intervention, with repeated-bout effect may have been a factor in modest increase in CK levels found. Further studies should therefore evaluate the effectiveness of HRW bathing during more intensive DOMS protocols while recruiting exercise naïve participants.

The present pilot trial demonstrated promising effects of supersaturated HRW bathing, yet several shortcomings have to be considered when interpreting the results of our study. We employed here a single session of HRW bathing which prevented us from evaluating the safety and efficacy of repeated baths and prolonged monitoring (>24 h) after HRV intervention. There may also be differences in the physiological effects of HRW baths among different protocols of exhaustive exercise and active populations, including various exercise modalities (such as running, cycling, or arm exercise), women, and/or age-specific groups. Besides, no information has been provided whether HRW effectively improves muscle damage biomarkers in elite athletes who regularly experience recurrent DOMS episodes throughout the training week or does HRW preserves the muscular performance of affected muscles in terms of power, strength, and endurance.

In conclusion, whole-body bathing in supersaturated HRW is a safe novel recovery protocol that attenuates muscular damage and can ease sore muscles after high-intensity eccentric exercise in active healthy men. The promising results of this pilot study should be corroborated and expanded in well-powered longitudinal trials with various athletic populations.

## Figures and Tables

**Table 1:** Changes in biomarkers of exercise-induced muscular damage (*n* = 6).

	Baseline	At 24-hour follow-up		*P* ^*∗*^
		HRW	Control water	
Creatine kinase (U/L)	343 ± 309	357 ± 189	465 ± 295^†^	0.04
Lactate dehydrogenase (U/L)	197 ± 36	188 ± 39	195 ± 38	0.74
Aldolase (U/L)	4.9 ± 1.8	4.6 ± 2.1	5.7 ± 2.4^†^	0.61
Aspartate transaminase (U/L)	27.5 ± 6.2	30.0 ± 8.0	32.3 ± 7.8^†^	0.58
Troponin I (mg/ml)	0.10 ± 0.02	0.10 ± 0.02	0.10 ± 0.02	0.99
Myoglobin (*μ*g/L)	53.5 ± 20.9	47.1 ± 21.8	43.5 ± 21.3	0.51
White blood cell count (10^9^/L)	4.6 ± 0.9	4.7 ± 0.8	5.0 ± 0.7	0.58
C-reactive protein (mg/L)	1.3 ± 1.5	0.9 ± 0.6	1.2 ± 1.2	0.74

Asterisk (*∗*) indicates *P* values from two-way ANOVA time *x* intervention for repeated measures (hydrogen-rich water (HRW) vs. control bath); ^†^sign indicates a significant difference between baseline vs. follow-up values at *P* < 0.05 for each intervention. Values are mean ± SD.

## Data Availability

The data used to support the findings of this study are available within the article.
